# A TRIM Family-Based Strategy for TRIMCIV Target Prediction in a Pan-Cancer Context with Multi-Omics Data and Protein Docking Integration

**DOI:** 10.3390/biology14070742

**Published:** 2025-06-22

**Authors:** Yisha Huang, Jiajia Xuan, Jiayan Liang, Xixi Liu, Yonglei Luo, Xuejuan Gao, Wanting Liu

**Affiliations:** MOE Key Laboratory of Tumor Molecular Biology and Key Laboratory of Functional Protein Research of Guangdong Higher Education Institutes, Institute of Life and Health Engineering, College of Life Science and Technology, Jinan University, Guangzhou 510632, China; yolololohuang@outlook.com (Y.H.); xuanjj@jnu.edu.cn (J.X.); roshell@stu2024.jnu.edu.cn (J.L.); xixiliu@stu2023.jnu.edu.cn (X.L.); yonglei1115@stu.jnu.edu.cn (Y.L.)

**Keywords:** TRIM family, PPI prediction, cancer, multi-omics, machine learning, protein docking

## Abstract

The TRIM CIV subfamily, distinguished by its C-terminal PRY-SPRY domains, constitutes nearly half of the human TRIM family and plays pivotal roles in cancer progression through ubiquitination. Identifying TRIM CIV substrates and interactors has emerged as a critical approach for elucidating tumorigenesis. Current protein–protein interaction (PPI) prediction models face challenges, including an inherent deficiency of negative datasets, biased feature integration, and the absence of a cancer-specific interaction context. To achieve the precise identification of TRIMCIV targets, we developed TRIMCIVtargeter with predictive models that systematically integrates multi-dimensional PPI features—expression differences and correlations in specific cancer, comparable protein-docking scores, and cancer-specific context. Learning from the functional and structural interaction features between 718 experimentally validated TRIM–target pairs, two types of SVM-based binary models were independently trained using proteomic and transcriptomic data. Our models achieved robust prediction performance in cancers utilizing a fair feature space and circumventing hypothetical non-interacting pairs. TRIMCIVtargeter not only provides a cancer-related resource for studying TRIMCIV-mediated regulatory mechanisms but also offers a new perspective for family-specific PPI prediction, holding significant implications for biomarker discovery and therapeutic targeting in oncology. The online platform of TRIMCIVtargeter is now available.

## Simple Summary

The E3 TRIM family plays a key role in cancer, but identifying which proteins they interact with is costly and challenging. Current prediction methods struggle because they rely on incomplete data or overlook how these interactions change in diseases like cancer. To solve this issue, we studied the largest TRIM subfamily (CIV), collecting hundreds of their known interactions from past experiments. We observed that CIV proteins consistently correlate with DEGs in cancers, unlike other TRIM members. Using this pattern—along with structural features and cancer-specific data—we built a computational tool called TRIMCIVtargeter to predict new CIV targets. Notably, our approach avoids artificial assumptions and accounts for disease context. This tool provides researchers with a faster, more accurate way to uncover TRIM-related cancer mechanisms, potentially accelerating the discovery of new therapeutic targets. By focusing on real-world biological trends, TRIMCIVtargeter advances our understanding of how these proteins contribute to cancer and offers a framework for studying other protein families in disease.

## 1. Introduction

The tripartite motif (TRIM) family has more than 70 members in humans, and it is classified into 12 subfamilies (CI to UC) on the basis of their domain organization, with the CIV subfamily accounting for nearly half of all members [1]. Most TRIM proteins contain a RING-finger domain at the N-terminus, which confers E3 ubiquitin ligase activity, while the C-terminal domains play crucial roles in recognizing substrate and interactors, collectively referred to as “targets” in this study [2,3]. The TRIM family has been extensively implicated in various cellular processes, particularly in carcinogenesis [4]. Notably, the CIV subfamily, which comprises over half of the TRIM family and is characterized by C-terminal PRY-SPRY domains, plays a key role in regulating cell progression through complex pathways across multiple cancer types [5,6]. The PRY-SPRY domain of the TRIM protein has been reported to serve as a critical component for substrate recognition and interactor binding in various biological pathways. For instance, TRIM21 interacts with GSDMD via its PRY-SPRY domain, stabilizing GSDMD expression in quiescent cells [7]. TRIM15 interacts with TAK1 and inhibits its K63-linked ubiquitination depending on its PRY-SPRY domain. [8] Similarly, the binding activity of TRIM25 also depends on its PRY-SPRY domain [9]. Current research efforts are focused on identifying potential targets of TRIM members through experimental approaches, which are essential for understanding tumorigenesis and discovering biomarkers in specific cancer contexts [2,10]. However, experimentally identifying TRIM-specific targets from the vast pool of cellular proteins is both labor-intensive and costly. Consequently, predictive models for protein–protein interactions (PPIs) became desirable to facilitate the identification of potential TRIM targets.

With the growing availability of biological data, computational approaches have been developed to predict PPIs based on the characteristics of interacting proteins, including sequence homology [11,12,13], co-expression patterns [14,15], Gene Ontology (GO) similarity [16,17], network topology [16,18], geometric features [19], and the physicochemical properties of binding regions [20,21]. Feature extraction and model construction have become mainstream methods in PPI prediction [22]. However, since only positive PPIs are well-documented in the literature, the lack of verified negative or non-interacting protein pairs poses a significant challenge in model training [23]. It is impractical to experimentally validate a comprehensive set of negative PPIs, and negative interaction databases such as Negatome provide very limited pairs, showing that they are barely available for model training [24]. Thus, researchers often resort to generating negative datasets based on hypotheses. EnPPIpred selected random protein pairs which are not reported in a negative dataset [25]. Zhang et al. generated non-PPI datasets based on low sequence similarity [26]. N Khunlertgit et al. considered the topological characteristic of interaction networks [27]. DPPN-SVM assumed non-interaction based on differential subcellular localization indirectly measured via co-expression and interaction networks [28]. However, these strategies introduce biases and limit model generalizability. For instance, it is widely acknowledged that proteins can translocate between organelles, such as mitochondria and chloroplasts, to participate in dynamic biological pathways [29,30]. Defining non-interacting protein pairs based solely on localization disregards these findings and can introduce significant biases.

Furthermore, biased features in protein–protein interaction increase dataset sparsity, ultimately compromising prediction accuracy. Liang et al. enriched GO terms and network topology in predictive models, which successfully improve the identification of well-studied “hub” proteins but still struggle to predict interactors for less-characterized proteins [16]. Current PPI prediction tools such as protein2vec [31], TANGO [32], and TransformerGO [33], which rely on GO annotation information, all exhibit inherent bias toward hub genes. Consequently, proteins outside of research hotspots often yield inaccurate predictions or cannot be predicted at all, particularly for novel proteins. An additional critical consideration is that protein interactions function within specific biological contexts, including tissue origin and disease phenotypes. However, most existing computational tools for PPI prediction tools, including docking programs, frequently overlook disease-specific factors in protein interactions, limiting their predictive accuracy in disease-related research.

Apart from physical interaction, functional interaction also presented a signal for protein interaction, assuming PPIs that display similar expression pattern are functionally associated [34]. The STRING database summarized predicted functional interactions of various organisms [35]. TRIM proteins mainly regulate cancer development through substrate ubiquitination, and many experiments also validated the co-expression pattern between TRIM members and their targets at the expression level [2]. For example, TRIM25 activated Nrf2 via ubiquitination-mediated Keap1 degradation, positively associated with Nrf2 expression and negatively with Keap1 expression in hepatocellular carcinoma [36]. Similarly, TRIM27 mediates the ubiquitination of TBK1 through SHP2 recruitment, showing a direct co-expression relationship with SHP2 [37]. In glioblastoma, a tissue microarray analysis of glioma samples revealed an inverse relationship between TRIM21 and TIF1γ expression levels, while TRIM21 was positively correlated with β-catenin levels [38]. The TRIM family has been identified as a key player in various cancers through interactions with differentially expressed genes (DEGs), which are widely recognized in tumorigenesis research and have been incorporated into model training such as cancer prediction [39] and overall survival prediction [40]. For example, TRIM59 is upregulated in gastric tumors to promote p53 degradation via ubiquitination [41], while TRIM31 competitively interacts with p53 in breast cancer, leading to its stabilization and activation [42]. In antiviral immune responses, ARRDC4 recruits TRIM65 to promote K63-linked ubiquitination of MDA5 [43]. These findings highlight DEGs as crucial TRIM targets in disease regulation. With the development of high-throughput methods, extensively available multi-omics data has been widely employed to elucidate the functional interaction of PPI in cancers. For example, Chen et al. investigated co-expression patterns between E3 ligases and their interacting substrates using pan-cancer multi-omics data and subsequently incorporated these features into their predictive model [44].

This study introduces a TRIM family-based methodology for protein–protein interaction (PPI) prediction. By systematically compiling and manually curating TRIM–target pairs from the literature, we leveraged the distinct and balanced representation of TRIMCIV and non-CIV subfamilies to develop binary classifiers capable of distinguishing TRIMCIV-specific interactions from the broader TRIM target pool. To construct a robust predictive framework, we integrated multi-omics data—including cancer proteomics and transcriptomics—with computational docking pipelines. An MS-based model leveraging cancer proteomic data and an RNAseq-based model utilizing transcriptomic data was trained and subsequently deployed in TRIMCIVtargeter, an online platform designed to facilitate the discovery of TRIMCIV targets across diverse cancer types. By providing a systematic resource for studying TRIM-mediated regulatory mechanisms in cancer, this work also presents a new avenue for PPI prediction.

## 2. Materials and Methods

### 2.1. TRIM–Target Interaction Database Construction

To construct a comprehensive TRIM–target interaction database, we retrieved publications focusing on Homo sapiens from the past decade using Google Scholar with the query keyword “TRIM* + ubiquitination.” Targets (substrates and interactors) of TRIM family members were manually curated based on key patterns found in the literature, including “TRIM* mediates/promotes/regulates the ubiquitination/degradation of TARGET”, “TRIM* targets TARGET for degradation”, “TRIM* interacts with/facilitates TARGET”, “TRIM*-TARGET ubiquitin signaling”, “TARGET is ubiquitinated and degraded by TRIM*”, and “K*-linked ubiquitination by TRIM* stimulates/regulates TARGET”. Here, TRIM* denotes a specific TRIM member, and TARGET refers to the corresponding substrate or interactor. The gene names of identified targets were standardized using the uniprot database (https://www.uniprot.org/, accessed on 16 June 2024). As a result, 718 unique TRIM–target pairs with 474 unique targets were manually curated to establish a high-confidence reference dataset.

### 2.2. Structural Alignment

TM-align is a structural alignment algorithm used to compare 3D protein structures based on their topology. A TM-score > 0.5 is recognized as a reliable indicator of significant structural similarity between proteins, as established by benchmark studies on protein structure alignment [45]. In this study, TM-align was employed to evaluate the structural similarity of C-terminal domains among TRIM family members. A TM-score > 0.5 was considered indicative of structural similarity, with higher scores reflecting greater structural resemblance.

### 2.3. Expression Data Collection, Processing, and Analysis

Protein expression datasets in specific cancer types were collected from supplementary data in publications. A total of 9 mass spectrometry (MS)-quantified datasets covering 8 cancer types (Samples *n* = 905) were selected, with each containing over 6000 proteins (Table S2). Due to the limited availability of MS datasets, we supplemented our analysis with transcriptomic data from the UCSC Xena database (TCGA, TARGET, and GTEx projects; https://xenabrowser.net/datapages/, accessed on 21 July 2024), where the study-specific biases of transcriptomic data were mitigated, enabling a comparative analysis between TCGA and GTEx [46]. The RNA profiles for proteins was also recommended as the strategy in testing the reliability of provided interactions [23]. We followed the same strategy recommended in [46] to cope with the insufficiency of normal samples. We obtained a total of 26 RNAseq datasets covering 24 cancer types (Samples *n* = 11,815; Table S2).

To identify differentially expressed genes (DEGs) at both the protein and RNA levels, gene names were standardized using the UniProt database. For the proteomics dataset, protein expression values were log_2_-transformed, and genes with >50% missing values across samples were excluded. Missing values were then imputed using the k-Nearest Neighbors (KNN) algorithm. For the transcriptomic dataset, low-count genes were filtered out using edgeR (v4.0.16), and expression matrices were normalized using the trimmed mean of M-values (TMM) method [47]. Only primary isoforms were retained, and duplicate gene expressions were averaged. DEGs were identified using limma (v3.58.1) with an adjusted *p*-value threshold of < 0.01 (FDR-corrected). Differential expression cutoffs were set at |logFC| > 0.5 for the MS-based model and |logFC| > 1 for the RNAseq-based model.

### 2.4. Correlation Analysis

In each cancer dataset, correlations between TRIM members and DEGs were assessed by spearmanr, with a significance threshold of *p* < 0.01. Overall, 17,336 significantly correlated DEGs and 71 TRIM members (including 36 from the CIV subfamily and 35 from other subfamilies) were selected for physical interaction analysis.

### 2.5. Docking Pipeline

Given that TRIM subfamilies are classified based on their C-terminal domains, we extracted C-terminal sequences of specific TRIM members using domain information from the PDB database. To evaluate TRIM–target binding affinity, we employed a ZDock-Rosetta-ZRank2 docking pipeline [48,49,50]. The performance of this docking pipeline has been assessed as a successful docking strategy with acceptable predictions by a communitywide experiment CAPRI project in multiple rounds, including rounds 6–11 [51], rounds 13–19 [52], and rounds 20–26 [53]. Target protein structures with Swiss-Prot-reviewed annotations were retrieved from the AlphaFold database (https://alphafold.ebi.ac.uk/, accessed on 1 August 2024). To prepare for docking, the full-length structures of the target and the C-terminal domains of TRIM members were marked. A small amount of PDB fails (~2%) in docking were attributed to overly large or technical errors. ZDock was run to generate 2000 conformations per TRIM-DEG pair. To include the hydrogen bond, Rosetta was used to add hydrogen atoms to the conformations, followed by ZRank2 scoring to rank out the best conformations. Each TRIM–target pair underwent the same evaluation workflow, receiving an interaction score (zrank_score) supported by the ZRank2 function, where lower scores indicate stronger binding affinity, and we assessed the interacting power including the Van der Waals energy, electrostatics energy, and desolvation. Given the computational demands of protein docking, we preprocessed TRIM-correlated DEGs and ultimately conducted docking for 364,068 TRIM-DEG pairs.

### 2.6. Predictor Building

To construct predictive models for TRIMCIV target identification, we first filtered reported TRIM–target pairs by selecting those with significant differential expression and correlation across different cancer datasets, as well as those successfully assessed for interaction by docking. The resulting dataset was divided into two groups: TRIMCIV–target pairs (labeled as TRUE) and TRIMelse–target pairs (TRIM member outside CIV subfamily and corresponding targets, labeled as FALSE). Due to differences in expression levels across datasets, we prepared two separate training datasets, respectively, for the MS-based model and the RNAseq-based model. These models aim to classify targets of TRIMCIV members.

To identify an optimal classification algorithm, we evaluated various machine learning models including Support Vector Machine (SVM), k-Nearest Neighbors (KNN), Linear Discriminant Analysis (LDA), Logistic Regression (LR), and Naïve Bayes (NB) and ultimately selected SVM due to its good performance. The SVM classifiers were fed with the logFC of target, correlation efficiency (R), disease type, and docking scores (zrank_score) between TRIM and target. Since expression data were derived from different disease backgrounds, fold change values for the same gene vary across different cancers. As SVM requires training data to be standardized, all numerical features were standardized before training.

Model training employed the scikit-learn python package (1.5.1), which utilizes the LIBSVM library as its backend [54]. The training datasets, consisting of binary labels (TRUE or FALSE), were mapped into a high-dimensional feature space via a kernel function. The optimal hyperplane was determined to maximize the margin between positive and negative instances. To enhance model performance, the optimal hyperparameter was found using grid search [55]. The final SVM models were implemented using a radial basis function (RBF) kernel, which showed robust sensitivity and specificity during validation. The decision function and kernel function were defined as follows:

(1) $$\begin{array}{c} f\left(x\right)=\sum_{i\in SV}y_{i}\alpha_{i}K\left(x_{i},x\right)+b \end{array}$$

(2) $$\begin{array}{c} K\left(x_{i},x_{j}\right)=\exp\left(-\frac{|x_{i}-x_{j}{|}^{2}}{2\sigma^{2}}\right)=\exp\left(-\gamma |x_{i}-x_{j}{|}^{2}\right) \end{array}$$

In the formula, $y_{i}$ denotes the class label of the support vector (with TRUE and FALSE mapped to 1 and 0, respectively). The coefficients $\alpha_{i}$ are obtained from the optimization process, and b represents the bias term. The kernel function $K(x_{i},x_{j}$) is the RBF kernel, where $x_{i}$ and $x_{j}$ are feature vectors. $\gamma =-\frac{1}{2\sigma^{2}}$ is a hyperparameter that controls the width of the RBF kernel. A larger γ value results in a narrower kernel, emphasizing local data points, whereas a smaller γ value leads to a smoother decision boundary. In this study, the squared Euclidean distance between them is given by

(3) $$\begin{array}{c} |x_{i}-x_{j}{|}^{2}={\left({\mathrm{disease}}_{i}-{\mathrm{disease}}_{j}\right)}^{2}+{\left(\log FC_{i}-\log FC_{i}\right)}^{2}+{\left(R_{i}-R_{j}\right)}^{2}+{\left({\mathrm{zrank}\text{\_}\mathrm{score}}_{i}-{\mathrm{zrank}\text{\_}\mathrm{score}}_{j}\right)}^{2} \end{array}$$

In SVM-based model training, the regulation factor, C, was used to control the trade-off between achieving a low training error and a low testing error, effectively regularizing the model to prevent overfitting. In this study, the MS-based model and RNAseq-based model were both trained with regulation factor C = 1 and γ = 1 after grid search for optimal hyperparameters.

Model training was generally conducted in a two-step process using a stratified shuffled fivefold cross-validation scheme [56]. First, a grid search was performed to optimize the hyperparameter of the SVM via fivefold cross-validation. The dataset was reshuffled, and hyperparameters were applied to model training and evaluation using fivefold cross-validation. Finally, the binary classifiers were retrained on the full dataset using the optimal hyperparameters before deployment.

### 2.7. Evaluation of Model Performance

To assess model performance, we used the Receiver Operating Characteristic (ROC) curve, which evaluates sensitivity (precision, PRE) and specificity (SPE) across different classification thresholds. The classification metric also included accuracy (ACC), recall (REC), F1 score (F1), and Matthews Correlation Coefficient (MCC), defined as follows:

(4) $$\begin{array}{c} \mathrm{PRE}=\frac{\mathrm{TP}}{\mathrm{TP}+\mathrm{FP}} \end{array}$$

(5) $$\begin{array}{c} \mathrm{SPE}=1-\frac{\mathrm{FP}}{\mathrm{FP}+\mathrm{TN}} \end{array}$$

(6) $$\begin{array}{c} \mathrm{ACC}=\frac{\mathrm{TP}+\mathrm{TN}}{\mathrm{TP}+\mathrm{FN}+\mathrm{TN}+\mathrm{FP}} \end{array}$$

(7) $$\begin{array}{c} \mathrm{REC}=\frac{\mathrm{TP}}{\mathrm{TP}+\mathrm{FN}} \end{array}$$

(8) $$\begin{array}{c} F1=\frac{2\times \mathrm{PRE}\times \mathrm{REC}}{\mathrm{PRE}+\mathrm{REC}} \end{array}$$

(9) $$\begin{array}{c} \mathrm{MCC}=\frac{\mathrm{TP}\times \mathrm{TN}-\mathrm{FP}\times \mathrm{FN}}{\sqrt{\left(\mathrm{TP}+\mathrm{FP}\right)\left(\mathrm{TP}+\mathrm{FN}\right)\left(\mathrm{TN}+\mathrm{FP}\right)\left(\mathrm{TN}+\mathrm{FN}\right)}} \end{array}$$

where TP and FP represent the numbers of true and false positives, while TN and FN are the numbers of true and false and negatives.

To evaluate model generalization, fivefold cross-validation was performed. The dataset was stratified into five approximately equal subsets, with four subsets used for training and the remaining subset used for testing. This process was repeated five times, ensuring that each subset served as a test set once. The numbers of TPs and FPs were averaged across the five iterations to compute the overall TP/FP ratio, as well as the final sensitivity and specificity values used for ROC analysis.

### 2.8. Web Interface Building

For the rapid screening of TRIM–target interactions, we developed an interactive web platform using Vite, Vue3, and ExpressJs. The platform integrates preprocessed datasets of TRIM–differentially expressed gene (DEG) pairs across 26 cancer types, with comprehensive metadata available on the data page. Our MS-based model comprises 79,720 high-confidence TRIM–target pairs, while the RNAseq-based model expands coverage to 1,356,496 putative interactions.

To assist users in selecting the candidates from multiple predicted interactions, we implemented ZRscore, a projection-based ranking metric that integrates both structural and functional evidence. This composite score combines normalized protein docking affinity scores (zrank_score) with co-expression correlation coefficients (R) through a geometric transformation. Each data point $\left(Zrankscore_{norm_{i}},R\right)$ undergoes normalization where zrank_score values are scaled to a [0, 1] range, denoted as $Zrankscore_{norm_{i}}$. These normalized docking scores are then paired with their corresponding co-expression values and projected onto a reference line defined by the equation $R=2Zrankscore_{norm_{i}}$ within a two-dimensional coordinate space, defined as

(10) $$\begin{array}{c} Z{\mathrm{Rscore}}_{i}=\frac{2Zrankscore_{norm_{i}}+\left|R_{i}\right|}{\sqrt{5}} \end{array}$$

A higher ZRscore indicates a more confident candidate. This projection approach, analogous to Principal Component Analysis (PCA), emphasizes the combined contribution of both features while maintaining their relative weights.

## 3. Results and Discussions

### 3.1. TRIM Family Overview and Reported TRIM–Target Pair Database Construction

**Figure 1:**
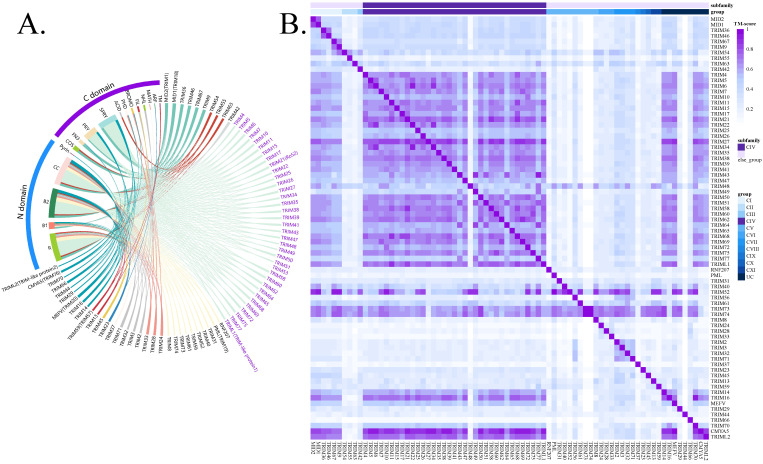
Overview of TRIM family and TRIMCIVtargeter. (**A**) Classification of TRIM family members based on C-terminal domain composition; TRIM CIV members are shown in purple. (**B**) Structural alignment of TRIM C-termini assessed by TM-align, with TM-score > 0.5 indicating significant similarity.

We first constructed the human TRIM family landscape based on domain composition and observed that TRIMCIV constitutes approximately 50% (36/76) of the TRIM family members (Figure 1A). The TRIMCIV group is characterized by a PRY-SPRY domain at the C-terminus, a critical component mediating protein–protein interaction, particularly in immune signaling pathways [1,6,57]. To further confirm the structural similarity of the C-terminus within the TRIM family, we conducted pairwise structural alignments using TM-align in bidirectional comparisons, ensuring a comprehensive and accurate assessment of structural similarity (Table S1). The results demonstrate that TRIMCIV members share highly similar C-terminal domains (overall TM-score > 0.5), distinguishing them from other TRIM groups (Figure 1B). While some TRIM family members (TRIM14, TRIM16, CMYA5, and TRIML2) in the uncategorized group (UC group) also contain the PRY-SPRY domain in their C-terminal region and their functional domain in the UC group has yet to be well characterized, we excluded these members to minimize interference in the identification of TRIMCIV targets in subsequent analyses.

**Figure 2:**
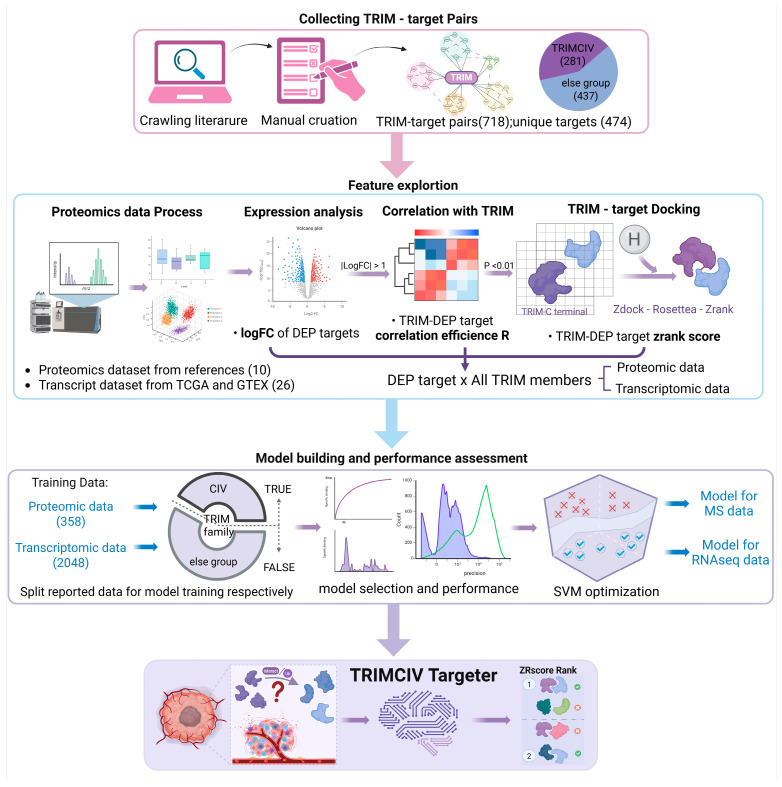
The framework of TRIMCIVtargeter. Reported TRIM targets were manually curated from the literature, and unique TRIM–target pairs were analyzed based on expression correlation and physical interactions via docking. Features including fold change, correlation coefficient (R) in specific cancer types, and interaction assessment (zrank_score) were integrated into the feature space for SVM-based model training. Two independent models were trained using gene expression in proteomics and transcriptomics, ultimately forming the TRIMCIVtargeter platform.

As the framework of this study (Figure 2), we first curated reported substrates and interactors and constructed a TRIM–target interaction database, containing 281 pairs within the TRIMCIV group and 437 pairs in other TRIM groups, with a total of 474 unique targets (Table S1). The TRIM–target pair database is also publicly accessible at https://bioinformaticsscience.cn/trimcivpred/#/trim-ref (accessed on 16 June 2024). These TRIM–target pairs were subsequently analyzed using proteomics and transcriptomic datasets to investigate their differential expression levels and correlations across various cancer types, as well as their physical interactions. These features constituted the dimensions of TRIM–target pairs and were subsequently utilized to train the two models based on the source of the expression data. The trained models were then integrated into the TRIMCIVtargeter public platform for application.

### 3.2. Distinct Correlation of TRIMCIV with DEG

**Figure 3:**
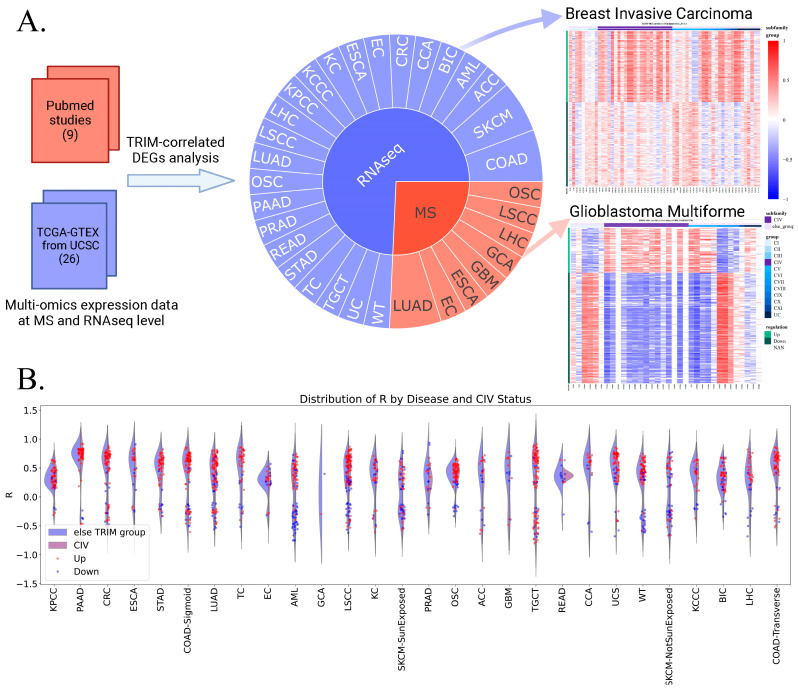
The correlation between TRIM proteins and DEGs across cancers. (**A**) A total of 9 MS datasets and 26 RNAseq datasets covering 26 cancer types were utilized to analyze correlations between TRIM proteins and differentially expressed targets, revealing distinct clustering patterns within the TRIMCIV group. (**B**) The R distribution between TRIM and targets across datasets. The cancer types include Adrenocortical Cancer (ACC), Acute Myeloid Leukemia (AML), Breast Invasive Carcinoma (BIC), Cholangiocarcinoma (CCA), Colon Adenocarcinoma (COAD), Colon and Rectal Cancer (CRC), Uterine Corpus Endometrioid Carcinoma (EC), Esophageal Carcinoma (ESCA), Kidney Chromophobe (KC), Kidney Clear Cell Carcinoma (KCCC), Kidney Papillary Cell Carcinoma (KPCC), Liver Hepatocellular Carcinoma (LHC), Lung Squamous Cell Carcinoma (LSCC), Lung Adenocarcinoma (LUAD), Ovarian Serous Cystadenocarcinoma (OSC), Pancreatic Adenocarcinoma (PAAD), Prostate Adenocarcinoma (PRAD), Rectum Adenocarcinoma (READ), Skin Cutaneous Melanoma (SKCM), Stomach Adenocarcinoma (STAD), Thyroid Carcinoma (TC), Testicular Germ Cell Tumor (TGCT), Uterine Carcinosarcoma (UC), Wilms Tumor (WT), Endometrial Carcinoma (EC), Glioblastoma multiforme (GBM), Gastric cancer (GCA), and High-Grade Serous Ovarian Carcinoma (OSC).

We analyzed multi-omics expression data across 26 cancer types, including 8 MS datasets from supplementary files of publication and 26 RNAseq datasets from the UCSC database. Differentially expressed genes (DEGs) were identified from each dataset (with an adjusted *p*-value threshold of <0.01 and |logFC| > 1) (Figure 3A; Table S2). Significantly expressed targets were filtered to examine the expression correlation with TRIM proteins across different cancer types (Figure 3B; Table S3). Our analysis of TRIM protein expression correlations across various cancer types revealed distinct patterns, particularly within the TRIMCIV family. As individual TRIM proteins typically interact with different targets, there are rare cases where multiple TRIMs (both CIV and non-CIV members) bind the same protein. Nevertheless, our co-expression analysis demonstrated that TRIMCIV members exhibit consistent correlation trends with DEGs across multiple cancer types. For instance, in the correlation landscape of breast invasive carcinoma and glioblastoma multiforme, TRIMCIV proteins exhibited a positive correlation with upregulated genes and a weaker correlation with downregulated genes (Figure 3A). More details of the co-expression relationship are included in Table S3. While these trends were not equally pronounced across all 35 cancer types analyzed, the recurrent correlations observed in multiple datasets suggest biological relevance among TRIMCIV members, supporting their utility for model construction.

### 3.3. Interaction Scoring via Protein Docking

**Figure 4:**
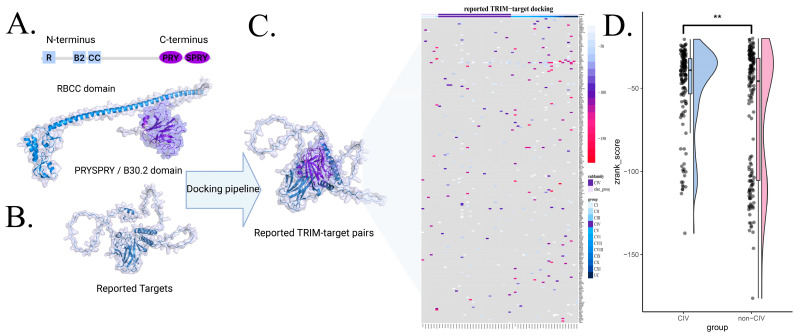
The schematic of docking reported TRIM–target pairs with the following pipelines: zdock was used to find the conformation, and Rosetta was used to add the hydrogen of conformation for ZRank2 to assess the best formation with the interaction score. (**A**) TRIMCIV structural model (e.g., TRIM21, P19474). TRIMCIV proteins exhibit a conserved domain architecture, with an N-terminal RING-finger domain, one or two zinc-finger B-boxes (B1 and B2), and a coiled-coil region, while the C-terminal PRY-SPRY/B20.3 domain is implicated in substrate recognition. (**B**) An example of a target structure (e.g., P53 and P04637). (**C**) Physical interactions of reported TRIM-target pairs assessed using the docking pipeline. Targets were filtered based on significantly differential expression and significant correlation with TRIM members. (**D**) The interacting score distribution of reported TRIM–target pairs. ** *p* < 0.01.

**Figure 5:**
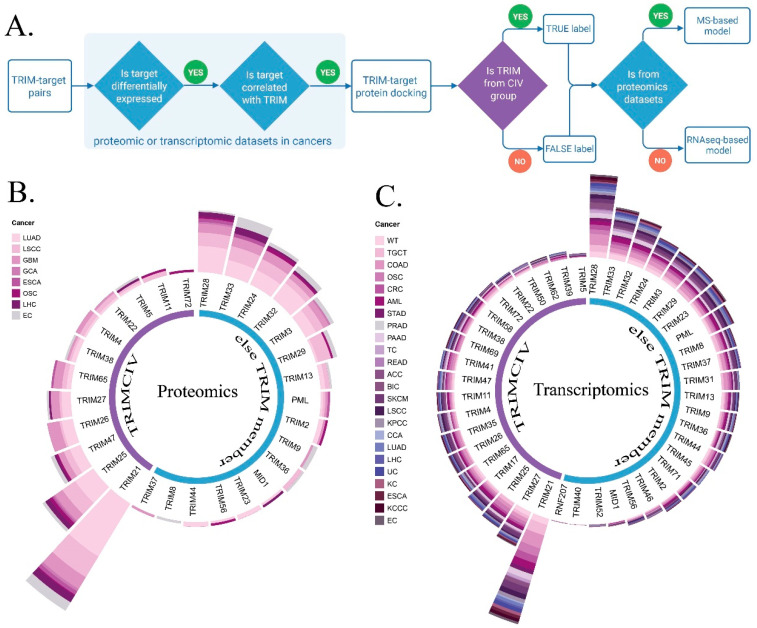
Dataset generation from proteomic and transcriptomic analyses across cancer types. (**A**) Workflow for generating and processing training datasets used to construct proteomics-based (MS) and transcriptomics-based (RNAseq) predictive models. (**B**,**C**) Composition of TRIM–target pairs identified in (**B**) proteomic and (**C**) transcriptomic datasets across different cancer types.

While the co-expression pattern of TRIM interactions does not necessarily indicate the physical interaction, we carried out protein docking to provide a more reliable measurement of their physical interactions [58]. To evaluate TRIM–target interactions, we filtered 244 reported targets based on significantly differential expression and significant correlation with TRIM proteins in cancers. The C-terminal domains of TRIM members were isolated and docked against their reported targets 2000 times (Figure 4A,B; Table S1). Protein docking was performed using a recommended pipeline integrating ZDOCK for initial docking, Rosetta for hydrogen addition, and ZRank2 for interaction scoring [48]. Although TRIMCIV proteins share a similar PRY-SPRY domain, the limited reported interactions and variability in the C-terminal domain pose challenges in definitively distinguishing TRIMCIV from other TRIM groups (Figure 4C; Figure S1). This docking protocol has been widely applied and assessed as an acceptable strategy by CAPRI [51,52,53], and its reliability was further examined, with sampled complexes consistently localizing near the PRY-SPRY domain of TRIMCIV proteins, thereby confirming biologically plausible binding poses (Figure S2). Further analysis revealed distinct docking score distributions between TRIMCIV and non-CIV TRIMs. The CIV group predominantly exhibited interaction scores in the range of −20 to −80, whereas non-CIV TRIMs displayed a more uniform score distribution (Figure 5; Wilcoxon test, ** *p* < 0.01). This suggests that the PRY-SPRY domain of TRIMCIV proteins tends to engage targets with moderate to strong binding power (around −40), though structural variations in interacting partners may also influence binding strength. To ensure comparability, each TRIM–target pair was processed through the same pipeline, enabling the evaluation of different TRIM proteins with the same target or different targets with the same TRIM protein. Here, we utilized docking scores as a quantitative feature for TRIM–target interactions to facilitate further model exploration.

### 3.4. Evaluation of Prediction Model

Based on our investigation and computational analysis, the reported TRIM–target pairs were characterized using multi-omics cancer data, including target protein logFC values, correlation coefficients (R), disease types, and physical interaction scores (zrank_score). Pairs were filtered using the following thresholds: logFC > 0.5 in proteomics or logFC > 1 in transcriptomics (adjusted *p* < 0.01, FDR), along with significant expression correlation (R, *p* < 0.01). The protein pairs were then divided into TRUE and FALSE datasets for model training and evaluation, employing shuffle cross-validation (Figure 4A). Ultimately, we compiled proteomic data (8 cancer types, *n* = 440) and transcriptomic data points (24 cancer types, *n* = 4438) for predictive modeling (Figure 5B,C). Due to both research bias (with TRIM21, TRIM25, and TRIM28 having more reported substrates and interactors) and our selection criteria (logFC, R, and *p*-value cutoffs), the TRIM–target pairs were unevenly distributed across TRIM proteins. However, since TRIM labels and their targets were excluded from the feature space during model training, this imbalance had a minimal impact on model performance.

**Figure 6:**
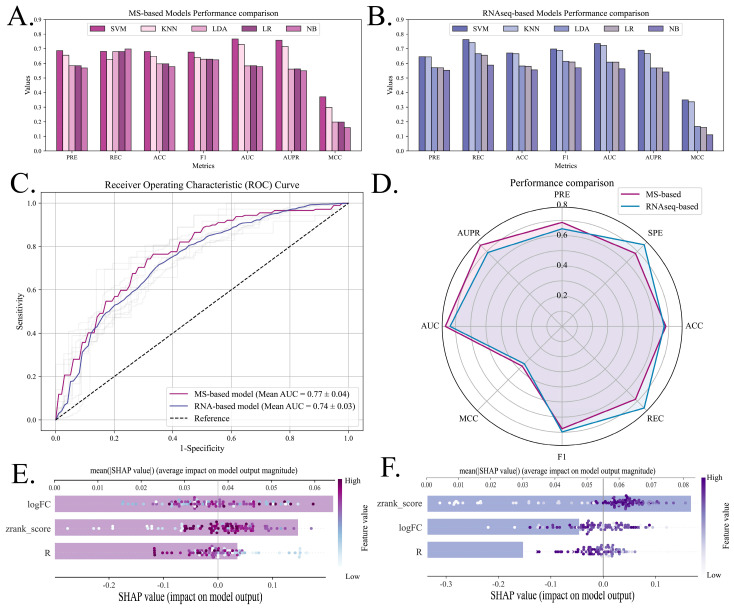
Performance evaluation of MS-based and RNAseq-based predictive models. (**A**,**B**) Performance comparison of five candidate machine learning algorithms (Support Vector Machine, k-Nearest Neighbors, Linear Discriminant Analysis, Logistic Regression, and Naïve Bayes) for (**A**) MS-based and (**B**) RNAseq-based models. (**C**,**D**) Receiver Operating Characteristic (ROC) curves averaged across five iterations of fivefold cross-validation, with overall performance metrics comparing (**C**) MS-based and (**D**) RNAseq-based models. (**E**,**F**) Combination of feature density scatter plot and important bar plot with SHAP to interpret MS-based model and RNAseq-based model, respectively. Lower abscissa represents SHAP value (lighter dots represent higher eigenvalues and vice versa), while upper *x*-axis represents feature importance scores in development of model.

Five candidate machine learning algorithms were selected to develop models predicting TRIMCIV targets in high-dimensional feature spaces constructed from log2-fold change (logFC) values, TRIM-DEG correlation coefficients (R), interaction scores (ZRank_scores), and cancer types by hot coding. In both MS data and RNAseq data, SVM outperformed KNN, LDA, LR, and NB in various metrics evaluated using fivefold cross-validation (Figure 6A,B). Therefore, the SVM was optimized using grid search to construct an MS-based model and an RNAseq-based model. The Receiver Operating Characteristic (ROC) curves of both models achieved robust performance, with an area under the ROC curve (AUC) of 0.77 in the MS-based model and 0.74 in the RNA model (Figure 6C; Table 1). In a comparative analysis, the performance of the MS-based model and the RNAseq-based model appears to be similar. However, the MS-based model exhibits a slight advantage, indicating the better predictive capacity of the MS-based model for TRIMCIV targets when leveraging features derived from proteomics data (Figure 6D). As proteomic data directly reflects protein-level expression and physical interactions, the MS-based model should offer higher confidence in cases where the target proteins are detectable by mass spectrometry.

However, considering the limited availability of proteomics data for target features and the potential utility of RNA-level expression data, both models were retained to ensure comprehensive and confident predictions. To further interpret the models, we utilized SHAP to illustrate the important features in the MS-based model and RNAseq-based model, respectively. The results show that logFC, R, and zrank_score were the top three dimensions contributing to the predictive model, which aligns with the biological knowledge underlying the feature selection process, validating the logical coherence of the feature set in capturing TRIM–target interactions (Figure 6E,F).

### 3.5. Web Server and Utility

**Figure 7:**
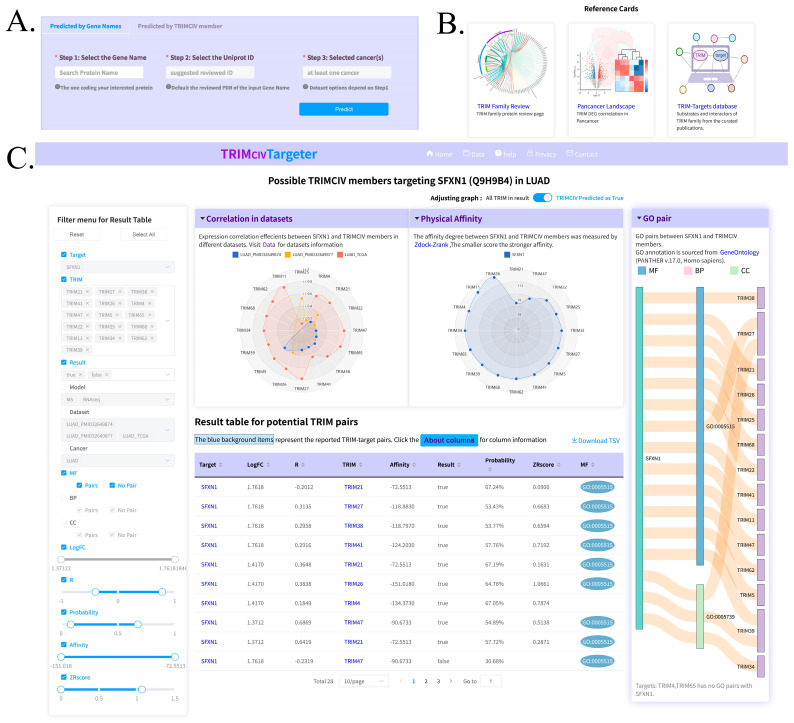
Overview of TRIMCIVtargeter platform. (**A,B**) Query interface and reference information of TRIMCIVtargeter designed to continuously integrate and learn from experimental validation data. * represents required input. (**C**) Result page displaying predicted TRIMCIV–target pairs and GO term associations between TRIM proteins and candidate targets.

To enable efficient access to TRIMCIV target predictions, we developed TRIMCIVtargeter, an interactive online platform (http://bioinformaticsscience.cn/trimcivpred/, accessed on 16 June 2024). Given the computational demands of protein docking, we pre-docked widely expressed TRIMCIV members against 13,091 differentially expressed human genes (at both the protein and RNA levels). The platform integrates preprocessed TRIM-DEG interaction data spanning multiple cancer types, comprising 79,720 pairs from the MS-based model and 1,356,496 pairs from the RNAseq model. Users can query potential interactions by gene name (with UniProt ID auto-completion for Swiss-Prot reviewed structures), select specific TRIMCIV members and cancer types, and retrieve candidate pairs predicted by either the MS-based model, RNAseq-based model, or both (Figure 7A). We also provided the TRIM family overview, landscape of pan-cancer datasets and manually curated reported TRIM-target information (Figure 7B). The results include detailed features for each TRIM–target pair, along with a ZRscore for candidate prioritization based on integrated R and zrank_score metrics (Figure 7C). A higher ZRscore indicates a more confident result. Other columns are also sortable for users to rank the candidate by their preferred features. While Gene Ontology (GO) terms were excluded from model training to avoid bias, the platform provides GO term overlap analysis between TRIM proteins and candidate targets as supplementary biological context (Figure 7C).

## 4. Conclusions

This study systematically identified TRIM family targets by comprehensively analyzing reported interactions. Current PPI prediction models face inherent limitations, including the inherent scarcity of non-interaction data, feature selection biases, and a lack of disease-specific contextualization. To overcome these challenges, we developed a targeted computational approach focusing on specific subfamilies within the broader TRIM family framework. Our comprehensive characterization revealed that the CIV subfamily represents both a biologically and computationally optimal choice for several reasons: First, it constitutes the largest TRIM subgroup (36/76 members in humans) and contains a distinctive, conserved PRY-SPRY domain at the C-terminus—a well-documented protein interaction module that mediates target recognition. Second, our systematic literature curation showed that CIV members account for over one-third of all experimentally validated TRIM–target interactions (281/718; Figure 2), providing substantial training data while maintaining balanced class distributions critical for machine learning applications. The biological relevance of the CIV subfamily-focused approach was further supported by multi-omics analyses. The integration of cancer transcriptomics and proteomics data revealed generally consistent TRIM-DEG correlation patterns among CIV members across multiple cancer types, indicating the relevance of biological function among the CIV subfamily. These observations align with the established structure–function relationships in protein interaction networks. As functional correlation does not necessarily indicate direct interaction, docking programs were implemented to further measure the physical interaction of TRIM–target pairs.

To construct a model for identifying the target of the CIV subfamily, we integrated multiple layers of biological evidence into a unified feature space, including cancer-specific fold changes in targets, expression correlations, and structural interaction measured by docking pipelines. According to different cancer data sources, we developed an MS-based model and an RNAseq-based model with particular attention to incorporating carefully validated true negative TRIMCIV–target pairs. Model evaluation confirmed confident predictive accuracy in identifying bona fide TRIMCIV targets. To bridge computational predictions with experimental research, we implemented these models in TRIMCIVtargeter, an interactive online platform that prioritizes high-confidence targets for experimental validation. This resource empowers researchers to efficiently explore TRIMCIV interactors and substrates implicated in cancer pathways. As multi-omics datasets and validated TRIMCIV–target interactions continue to expand, we anticipate further refinement of prediction accuracy through iterative model optimization.

Notably, rather than relying on hypothetical negative PPIs, TRIMCIVtargeter utilizes carefully curated positive and negative datasets derived from experimentally validated TRIM–target interactions in cancer. This approach ensures a balanced feature space while overcoming the common challenges of data sparsity and feature bias typically associated with hub proteins. However, this study also faces limitations. The model’s performance may be constrained by the currently limited TRIM family PPI data and proteomics coverage. While our transcriptomic analysis included 24 cancer types, MS-based proteomic modeling was restricted to 8 types due to data availability. The next version should include improvements by expanding omics datasets, developing more robust feature engineering approaches, and implementing advanced model architectures to better capture subfamily interaction differences and enhance predictive performance. Furthermore, as the first dedicated PPI predictor for a specific protein family, TRIMCIVtargeter demonstrates robust performance while occupying a unique methodological niche, which also presents challenges for this work to compare with existing models.

In this study, the reported targets of TRIM family proteins contain two types: canonical substrates undergoing ubiquitination (leading to either degradative or non-degradative outcomes) and binding partners involved in regulatory mechanisms. For example, TRIM21 interacts with PRMT5 (protein arginine methyltransferase 5), modulating TXNIP/p21 expression without inducing PRMT5 degradation [59]. The N protein of SARS-CoV-2 promoting TRIM25 interacts with G3BP2 (GTPase-activating protein SH3 domain–binding protein 2), inhibiting type I interferon production in the process of infection without involving the ubiquitination of TRIM25 [60]. As the first type of TRIM interaction only accounts for nearly half of the reported cases, the comprehensive prediction of TRIM-mediated PTM effects is particularly challenging. Furthermore, most existing studies focus on establishing protein–protein interaction networks in disease contexts rather than identifying precise modification sites—crucial information for understanding PTM hotspots. For instance, TRIM25-mediated ubiquitination accelerates RBPJ degradation via proteasome in bladder cancer validated by immunoprecipitation without further studying the binding residues [61]. In vitro and in vivo assays demonstrated that whereas TRIM23 ubiquitinated ANO1, leading to its stabilization, TRIM21 ubiquitinated ANO1 and induced its degradation [62], yet there is no detailed PTM information for reference. As research progresses, we anticipate the accumulation of more comprehensive PTM data for TRIM E3 ligases to enable more accurate predictions. Although our current model predicts interactions at the binding level, it provides researchers with preliminary interaction information for the TRIMCIV subfamily to guide subsequent experimental investigations.

In conclusion, this study introduced a new methodology for PPI prediction in the TRIM family and developed the TRIMCIVtargeter platform for predicting potential TRIMCIV–target interactions. This work provides valuable insights for investigating cancer-specific protein interactions and demonstrates the potential of family-specific modeling to overcome limitations in conventional PPI prediction methods. This platform provides an entry for the practical use of models and valuable information to assist users in experimental research.

## Figures and Tables

**Table 1:** Final models’ performance after being trained with optimized hyperparameters and balanced datasets.

Model	PRE	SPE	ACC	REC	F1	MCC	AUC	AUPR
MS-based	0.687 ± 0.051	0.682 ± 0.130	0.681 ± 0.046	0.682 ± 0.130	0.677 ± 0.068	0.371 ± 0.082	0.767 ± 0.046	0.758 ± 0.029
RNAseq-based	0.645 ± 0.020	0.763 ± 0.058	0.671 ± 0.022	0.763 ± 0.058	0.698 ± 0.026	0.350 ± 0.049	0.736 ± 0.032	0.690 ± 0.051

## Data Availability

TRIMCIVtargter is publicly available on the data page and reference cards of the following website https://bioinformaticsscience.cn/trimcivpred/ (accessed on 16 June 2024). The framework of the platform and model training process are available at https://github.com/yolololo-huang/TRIMCIVtargeter.git (accessed on 16 June 2024).

## Supplementary Materials

The following supporting information can be downloaded at: https://www.mdpi.com/article/10.3390/biology14070742/s1. Tables can be download at https://zenodo.org/records/14967126 (accessed on 16 June 2024). Figure S1: The TRIM–target pairs from reported interactions; Figure S2: Examples of TRIMCIV-target pair at different scores; Table S1: C-domain split information of TRIM, TMalign result of TRIM C domain, and reported TRIM–target pairs; Table S2: Source of multi-omics data and differential expressed genes across cancer types; Table S3: DEG-TRIM family-wide correlation across cancer types.
