# Supplementary material for: A TRIM Family-Based Strategy for TRIMCIV Target Prediction in a Pan-Cancer Context with Multi-Omics Data and Protein Docking Integration
Source: Biology (Basel). 2025 Jun 22;14(7):742. doi: 10.3390/biology14070742 (PMC12292072; doi:10.3390/biology14070742)
Supplement: Supplementary file 1 [file biology-14-00742-s001.zip › biology-3640033-supplementary.pdf]

TRIM−target interaction scoring

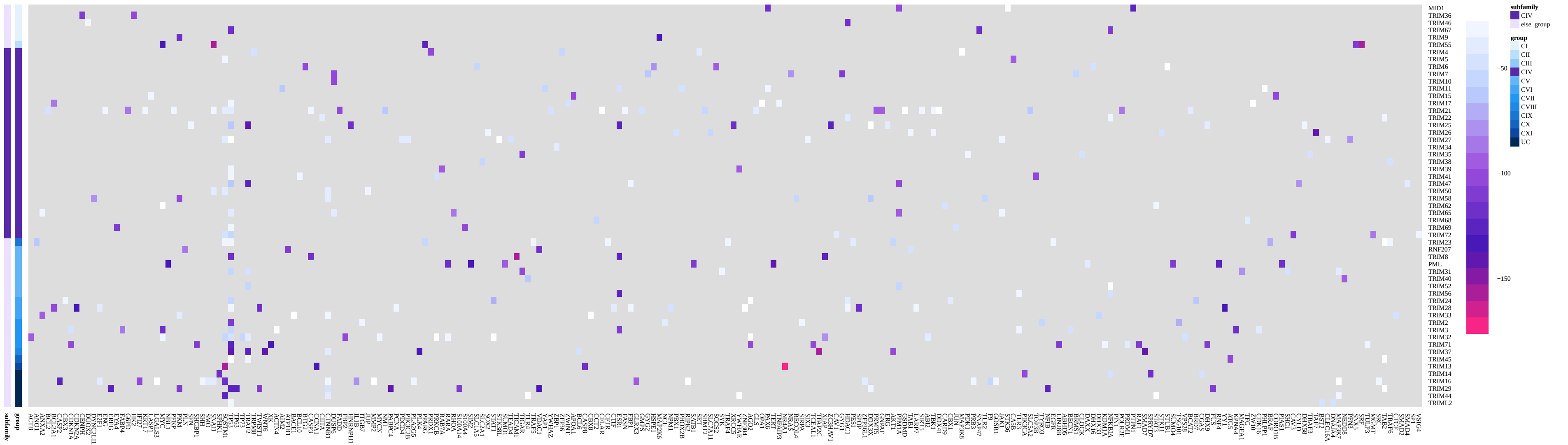

To further evaluate the docking pipeline's reliability, we sampled several reported TRIMCIV interactions and visualized complexes at varying score thresholds (**Figure S2**). Although we could not examine every predicted complex due to the scale of reported interactions, the sampled structures consistently showed that interacting proteins docked near the PRYSPRY domain of TRIMCIV, aligning with established findings that this domain is the primary interaction site. This supports the pipeline's accuracy in predicting biologically plausible binding poses.

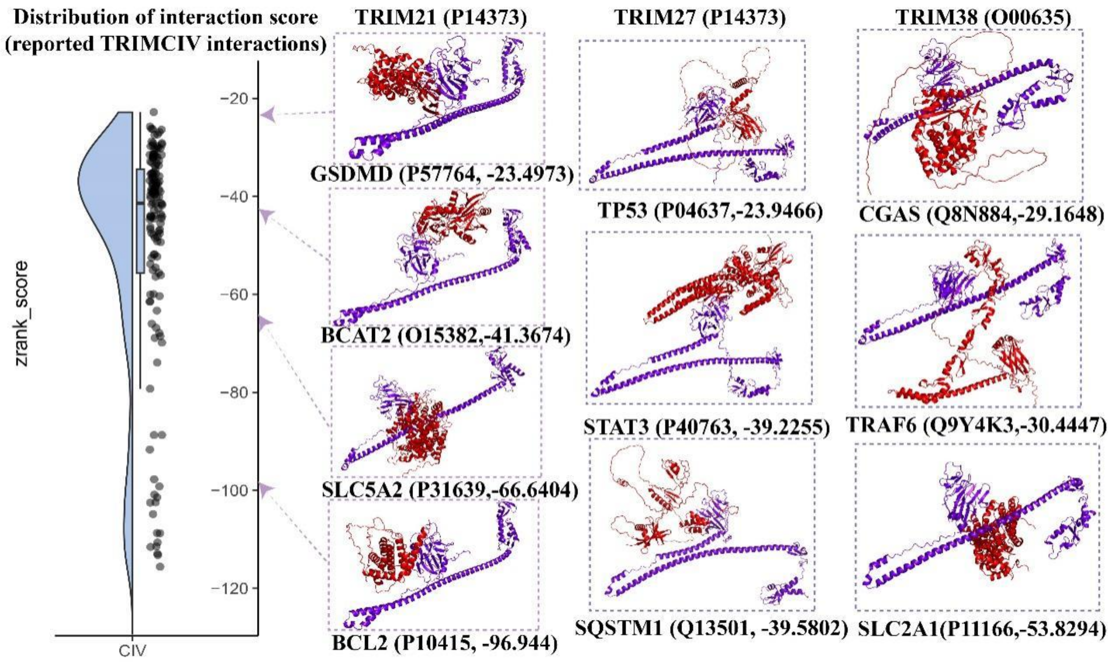

Figure S2. Examples of TRIMCIV-target pair at different scores
